# Programmed Death Ligand 1 (PD-L1) Expression in Epithelial Ovarian Cancer: A Comparison of Type I and Type II Tumors

**DOI:** 10.31557/APJCP.2019.20.4.1161

**Published:** 2019

**Authors:** Wilasinee Nhokaew, Pilaiwan Kleebkaow, Nipon Chaisuriya, Chumnan Kietpeerakool

**Affiliations:** 1 *Department of Obstetrics and Gynaecology, *; 2 *Department of Pathology, Faculty of Medicine, Khon Kaen University, Thailand. *

**Keywords:** Ovarian cancer, prognostic value, survival- PD-L1 expression, immunotherapy

## Abstract

**Objective:**

To examine the expression of programmed death ligand 1 (PD-L1) in type I and type II epithelial ovarian cancers (EOC) and its associations with outcomes.

**Methods:**

Records of 132 women with EOC were reviewed. Immunostaining of PD-L1 was performed with formalin-fixed, paraffin-embedded specimens. Expression of PD-L1 was classified into four categories (0; 1+; 2+; 3+) according to intensity of expression. Expression of PD-L1 ≥2+ was deemed to be high.

**Results:**

Of the 132 women, 75 (56.8%) and 57 (43.2%) women had type I and type II tumors, respectively. Approximately 70% of cases exhibited high PD-L1 expression. There was no significant difference in the rate of high PD-L1 expression between the two EOC types (65.3% versus 59.6%). In type I tumors, high PD-L1 expression was associated with more advanced stages (51.0% versus 34.6%), greater recurrence (46.9% versus 26.9%), and shorter median progression-free survival (27 months versus 62 months) than low expression. In type II tumors, there were no apparent differences between high and low expression of PD-L1 in terms of the percentage of advanced-stage tumors (82.6% versus 79.4%), recurrence (56.5% versus 58.8%), and median progression-free survival (21 months versus 24 months).

**Conclusion:**

high PD-L1 expression is associated with worse oncological outcomes in type I EOC. This finding emphasizes the merit of further studies to confirm this promising result and to determine the potential role of PD-L1 blockade therapy in type I EOC.

## Introduction

Epithelial ovarian cancer (EOC) is the most common type of ovarian cancer, accounting for more than 90% of all cases (Ledermann et al., 2013). Advances in the understanding of molecular pathogenesis have revealed two types of EOC. Type I EOC frequently presents as a large unilateral ovarian cyst. The common pathology of type I EOC includes low-grade serous carcinoma, clear cell carcinoma, endometrioid carcinoma, and mucinous carcinoma. The majority of women with type II EOC present when the disease is in its advanced stages. The most common pathology of type II EOC is high-grade serous carcinoma. Type II tumors account for approximately 90% of the deaths from EOC (Kurman and Shih Ie, 2016).

Systematic surgical staging for EOC is conducted to determine the extent of the disease and perform standard surgical treatment at the same time. Neoadjuvant chemotherapy (NACT) may be considered in women with advanced-stage EOC to minimize the potential of severe adverse events following debulking surgery (Morrison et al., 2012; Vergote et al., 2010) Adjuvant chemotherapy is indicated in the majority of cases with the aim of minimizing the risk of recurrence or prolonging amount of time to progression (Jaaback et al., 2016; Lawrie et al., 2015; Ledermann et al., 2013). However, the majority of women with EOC only temporarily respond to surgery and adjuvant chemotherapy, particularly those with advanced-stage tumors. Approximately 70% of these women experience cancer recurrence in the first three years (Ledermann et al., 2013). Therefore, future research investigating treatment for EOC that is effective in the long term is of utmost importance. 

In recent years, immunomodulation has come to play an important role in cancer treatment (Sharma and Allison, 2015). One promising method in this kind of treatment is the use of antibodies against programmed death ligand-1 (PD-L1), a novel class of immune checkpoint blockade (Alsaab et al., 2017). Physiologically, the programmed death-1 (PD-1) receptor is markedly expressed on the surface of activated T cells. Its ligands, PD-L1 and PD-L2, are commonly expressed on the surface of dendritic cells, macrophages, activated vascular endothelial cells, and mesenchymal stem cells. The main ligand of PD-1 is PD-L1 (Zhu and Lang, 2016). The interactions between PD-1 and its ligands lead to the inhibition of the function of cytotoxic T cells, thus limiting inflammatory response (Alsaab et al., 2017; Sharma and Allison, 2015; Zhu and Lang, 2016). However, some cancers may express PD-L1 and, as a result, circumvent the generation of tumor-induced immune suppression. Expression of PD-L1 on cancer cells is thought to represent classical adaptive immune resistance in cancer. PD-L1 inhibitors pharmacologically block the PD-1/PD-L1 interaction, thus enhancing immunologic response in destroying the cancer cells (Alsaab et al., 2017; Sharma and Allison, 2015). Examples of checkpoint blockades that target PD-L1 include atezolizumab, avelumab, pembrolizumab, and durvalumab (Alsaab et al., 2017).

PD-L1 inhibitors have been shown to be helpful in treating various types of cancer including metastatic melanoma, non-small cell lung cancer, and gastrointestinal cancer (Alsaab et al., 2017; Sharma and Allison, 2015). The overexpression of PD-L1 is a widely-explored predictive biomarker for the response to Anti-PD-L1 treatment (Alsaab et al., 2017; Zhu and Lang, 2016). In the present study, we assessed the expression of PD-L1 in type I and type II EOC and its associations with oncological outcomes. The findings of this study may identify a subset of cancers especially vulnerable to immune checkpoint therapy.

## Materials and Methods

*Study population*

This was a retrospective study, in which the medical records of women diagnosed with EOC who had been treated at Srinagarind Hospital, Khon Kaen, Thailand between January 2013 and December 2017 were reviewed. Baseline patient characteristics, detailed pathology results, stages of the disease, and survival outcomes were abstracted from the medical records. Surgical specimens from each patient were initially examined or reviewed by the gynecologic pathologist. The staging was updated according to the current International Federation of Gynecology and Obstetrics (FIGO) staging classification (Prat, 2014). Approval for the study was obtained from the hospital’s research ethics committee.

*Classifications of epithelial ovarian cancer*

The current model of ovarian carcinogenesis classifies EOC into two types (Kurman and Shih Ie, 2016). Type I tumors originate from benign extra-ovarian lesions that can transform into malignant lesions. Examples of type I tumors are endometrioid carcinoma, clear cell carcinoma, and seromucinous carcinoma, which are known to be associated with underlying endometriosis. Other less common histologies of type I tumors include low-grade serous carcinoma, mucinous carcinoma, and Brenner tumors. Type II tumors develop from intraepithelial lesions in the fallopian tube and are further classified into three groups: (i) high-grade serous carcinoma, (ii) carcinosarcoma, and (iii) undifferentiated carcinoma (Kurman and Shih Ie, 2016).

*Immunohistochemistry staining and interpretation of PD-L1 expression*

**Figure 1:**
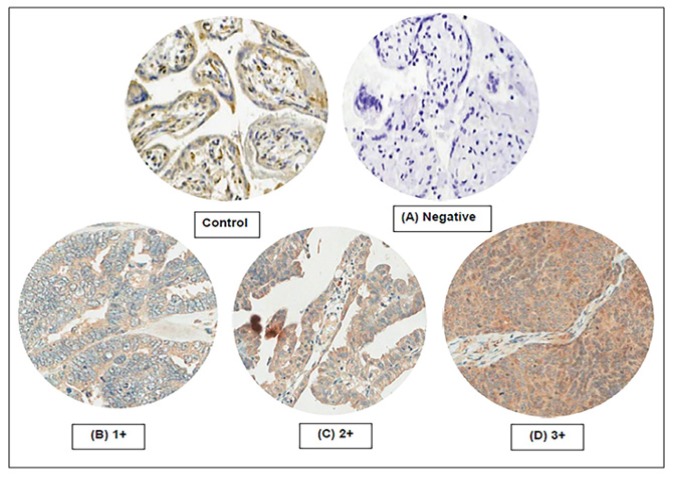
Staining Patterns of PD-L1. The control was normal placental tissue. Expression of PD-L1 was categorized as: (A) negative expression; (B) 1+ (positive expression but weaker than placenta); (C); 2+ (equivalent to expression in placenta): and (D) 3+ (stronger expression than placenta)

Hematoxylin and eosin (H&E) sections were re-evaluated to select tumor area. Areas containing fibrosis, adipose tissue or necrosis were avoided. Duplicate were punched from representative areas of a formalin-fixed paraffin embedded tissue block and re-embedded using the tissue microarray technique. Normal human placenta was also used as a positive control in the same block. Immunohistochemical staining was performed on sections (4 µm) from the microarray block using anti PD-L1 (clone 27A2) mouse monoclonal antibody (LifeSpan BioSciences, United States) at a dilution 1:200 on a Ventana- Benchmark XT autostainer. The expression of PD-L1 was categorized into four groups according to the intensity of the staining, as follows: 0 (negative expression), 1+ (positive expression but weaker than placenta), 2+ (equivalent to expression in placenta), and 3+ (stronger expression than in placenta). Positive stainings at immune cells and non-neoplastic areas were excluded. Assessment and scoring of PD-L1 expression were performed by two independent pathologists (PK and NC) who were unaware of the outcomes. Figure 1 represents the staining patterns of PD-L1 on ovarian cancers. Expression of PD-L1 ≥2+ was considered high. 

*Statistical analysis*

Descriptive statistics were used for baseline demographic data. The associations between the expression of PD-L1 and characteristics of the disease were analyzed via the χ^2^ or Fisher exact test, as appropriate. Disease-free survival was calculated using the Kaplan-Meier method and difference in survival was compared using the log-rank test. 

## Results

*Clinical profiles of the patients*

During the study period, 132 women with EOC were reviewed. The mean age of the women was 54.3 years (range, 25–81 years). Eighty-six (65.2%) of the women were multiparous and 47 (35.6%) were premenopausal. Seventy-five (56.8%) of the women were found to have type I tumors including clear cell carcinoma (39), endometrioid carcinoma (21), mucinous carcinoma (8), and low-grade serous carcinoma (7). Histology in the 57 women who had type II tumors revealed high-grade serous carcinoma. Forty (30.3%) women received NACT prior to definitive surgery. 

Table 1 presents the characteristics and clinical outcomes of patients stratified by type of tumor. Women with type II tumors were significantly older and more likely to present at later stages than those with type I EOC. At a median follow-up time of 16.5 months (interquartile range, 11-27 months), women with type II tumors had a significantly higher rate of cancer recurrence (57.9% versus 40.0%, respectively; P=0.041). There was no significant difference in the rate of high PD-L1 expression between type I and type II EOC (65.3% and 59.6%, respectively; P=0.503).

**Table 1:** Characteristics and Clinical Outcomes of Patients Stratified by Type of the Tumors

Variables	All patients (n=132)		Type of the tumors		P-value a
			Type I (n=75)	Type II (n=57)	
Age (years)					
	Mean ± SD	54.3±9.9	51.3±9.3	58.2±9.3	< 0.001
	≥ 60	43 (32.6)	16 (21.3)	27 (47.4)	0.002
	< 60	89 (67.4)	59 (78.7)	30 (52.6)	
Parity status					
	Nulliparous	46 (34.8)	34 (45.3)	12 (21.1)	0.004
	Multiparous	86 (65.2)	41 (54.7)	45 (78.9)	
Menopausal status					
	Premenopausal	47 (35.6)	34 (45.3)	13 (22.8)	0.007
	Postmenopausal	85 (64.4)	41 (54.7)	44 (77.2)	
Stages of disease					
	Stage I	38 (28.8)	32 (42.7)	6 (10.5)	< 0.001
	Stage II	14 (10.6)	9 (12.0)	5 (8.8)	
	Stage III	61(46.2)	28 (37.3)	33 (57.9)	
	Stage IV	19 (14.4)	6 (8.0)	13 (22.8)	
Largest tumor size in the ovaries (cm)					
	≥ 10	82 (62.1)	55 (73.3)	27 (47.4)	0.002
	< 10	50 (37.9)	20 (26.7)	30 (52.6)	
Receiving NACT		40 (30.3)	13 (17.3)	27 (47.4)	<0.001
Intensity of PD-L1 expression					
	1+	49 (37.1)	26 (34.7)	23 (40.4)	0.677
	2+	71 (53.8)	41 (54.7)	30 (52.6)	
	3+	12 (9.1)	8 (10.6)	4 (7.0)	
	High expression (≥ 2+)	83 (62.9)	49 (65.3)	34 (59.6)	0.503
Recurrence of disease		63 (47.7)	30 (40.0)	33 (57.9)	0.041
Platinum resistance ^b^		20 (15.2)	13 (17.3)	7 (12.3)	0.423

SD, standard deviation; NACT, neoadjuvant chemotherapy; PD-L1, programmed death ligand-1; a Comparing between type I and type II tumors; b, Occurring ≤ 6 months following adjuvant platinum chemotherapy.

**Table 2:** Expression of PD-L1 among Patients with Type I Tumors Stratified by Baseline Variables (n=75)

Variables		Intensity of PD-L1 expression		P-value
		Low expression a (n=26)	High expression b (n=49)	
Age (years)				
	Mean ± SD	50.5±9.5	51.7±9.2	0.599
	≥ 60	4 (15.4)	12 (24.5)	0.36
	< 60	22 (84.6)	37 (75.5)	
Parity status				
	Nulliparous	13 (50.0)	21 (42.9)	0.554
	Multiparous	13 (50.0)	28 (57.1)	
Menopausal status				
	Premenopausal	15 (57.7)	19 (38.8)	0.117
	Postmenopausal	11 (42.3)	30 (61.2)	
Stages of disease				
	Stage I-II	17 (65.4)	24 (48.9)	0.174
	Stage III-IV	9 (34.6)	25 (51.0)	
Largest tumor size in the ovaries (cm)				
	≥ 10	20 (76.9)	35 (71.4)	0.609
	< 10	6 (23.1)	14 (28.6)	
Receiving NAC		3 (11.5)	10 (20.4)	0.334
Recurrence of disease		7 (26.9)	23 (46.9)	0.092
Platinum resistance ^c^		2 (7.7)	11 (22.4)	0.108

SD, standard deviation; NACT, neoadjuvant chemotherapy; PD-L1, programmed death ligand-1; ^a^, Positive expression but weaker than placenta (1+); ^b^, Equivalent or stronger to expression in placenta (2+ and 3+); ^c^, Occurring ≤ 6 months following adjuvant platinum chemotherapy.

**Table 3:** Expression of PD-L1 among Patients with Type II Tumors Stratified by Baseline Variables (n=57)

Variables		Intensity of PD-L1 expression		P-value
		Low expression a (n=23)	High expression b (n=34)	
Age (years)				
	Mean ± SD	57.5±10.9	58.7±8.1	0.628
	≥ 60	11 (47.8)	16 (47.1)	0.955
	< 60	12 (52.2)	18 (52.9)	
Parity status				
	Nulliparous	6 (26.1)	6 (17.6)	0.443
	Multiparous	17 (73.9)	28 (82.4)	
Menopausal status				
	Premenopausal	6 (26.1)	7 (20.6)	0.627
	Postmenopausal	17 (73.9)	27 (79.4)	
Stages of disease				
	Stage I-II	4 (17.4)	7 (20.6)	0.764
	Stage III-IV	19 (82.6)	27 (79.4)	
Largest tumor size in the ovaries (cm)				
	≥ 10	13 (43.5)	14 (41.2)	0.255
	< 10	10 (56.5)	20 (58.8)	
Receiving NACT		14 (60.9)	13 (38.2)	0.255
Recurrence of disease		13 (56.5)	20 (58.8)	0.863
Platinum resistance c		4 (17.4)	3 (8.8)	0.334

SD, standard deviation; NACT, neoadjuvant chemotherapy; PD-L1, programmed death ligand-1, ^a^, Positive expression but weaker than placenta (1+); ^b^, Equivalent or stronger to expression in placenta (2+ and 3+); ^c^, Occurring ≤ 6 months following adjuvant platinum chemotherapy.

**Figure 2:**
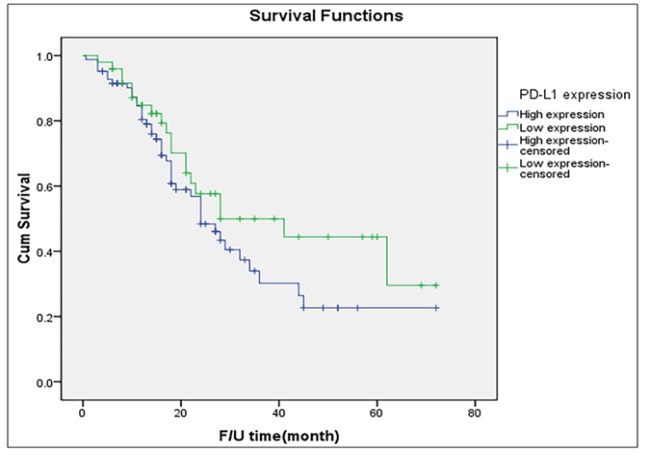
Disease-Free Survival of All Patients Stratified by Intensity of PD-L1 Expression (Log-Rank Test= P=0.172)

**Figure 3:**
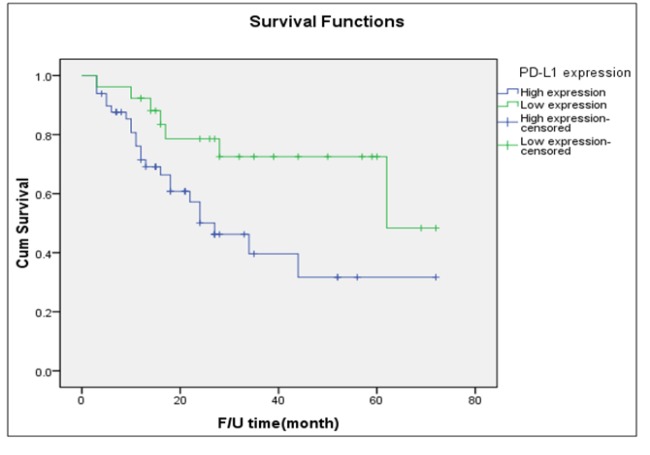
Disease-Free Survival of Patients with Type I Tumor Stratified by Intensity of PD-L1 Expression (Log-Rank Test =0.019)

**Figure 4:**
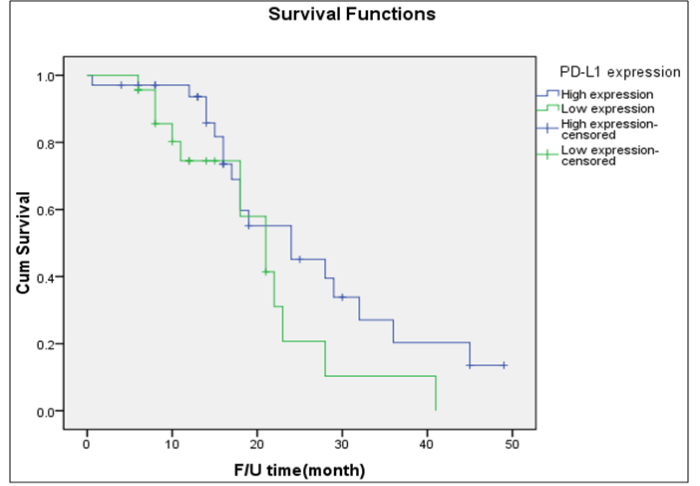
Disease-Free Survival of Patients with Type II Tumor Stratified by Intensity of PD-L1 Expression (Log-Rank Test = 0.108)

**Figure 5:**
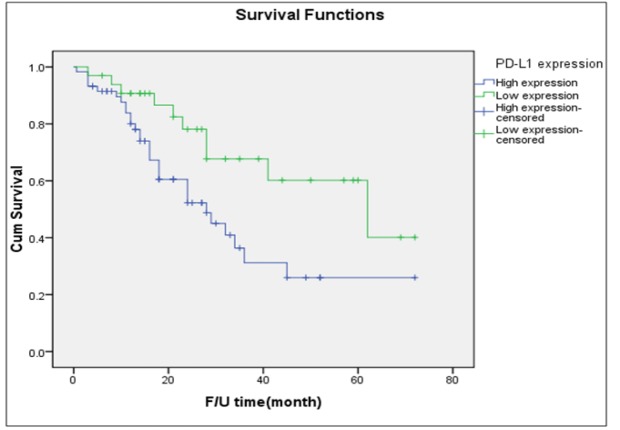
Disease-Free Survival of Patients Who Did not Receive Neoadjuvant Chemotherapy Stratified by Intensity of PD-L1 Expression (Log-Rank Test= 0.018)

**Figure 6:**
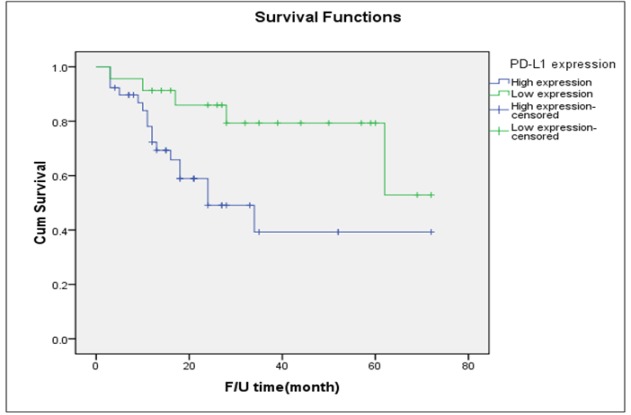
Disease-Free Survival of Patients with Type I Tumors Excluding Those Receiving Neoadjuvant ChemoTherapy, Stratified by Intensity of PD-L1 Expression (Log-Rank Test= 0.018)

**Figure 7:**
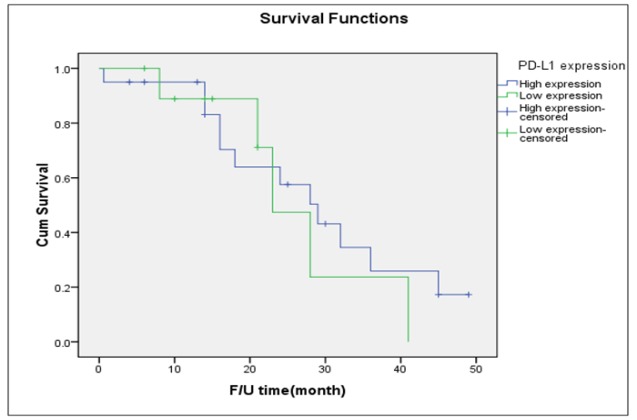
Disease-Free Survival of Patients with Type II Tumors Excluding Those Receiving Neoadjuvant ChemoTherapy, Stratified by Intensity of PD-L1 Expression (Log-Rank Test=0.566)

*PD-L1 expression and patient prognosis*

Table 1 shows the results of PD-L1 staining among the 132 women included in this study. The surgical specimens of 83 (62.9%) of the women were noted as exhibiting high PD-L1 expression. The distributions of the intensity of PD-L1 expression were almost identical across the two different types of tumors. Figure 2 presents the probability of survival among the entire cohort. The median PFS of women with high expression of PD-L1 was slightly lower than that noted among women with low intensity of expression (24 months versus 28 months). 

Table 2 displays the outcomes of women with type I tumors cross-tabulated by the intensity of PD-L1 expression. Of the 75 women with type I tumors, 49 (65.3%) had high PD-L1 expression. There were no significant differences between women with high PD-L1 expression and those with low expression in terms of age, parity status, or menopausal status. Women with high expression were more likely to present at later stages (51.0% versus 34.6%) and experience disease recurrence (46.9% versus 26.9%) than those with low intensity PD-L1 expression. Women with high expression of PD-L1 had a significantly shorter median PFS than those with low expression (27 months versus 62 months; Figure 3). 

Table 3 presents the outcomes of the 57 women who had high-grade serous carcinoma stratified by the intensity of PD-L1 expression. Thirty-four (59.6%) women were noted to have high PD-L1 expression. There were no significant differences between women with high PD-L1 expression and those with low expression in terms of patients’ age, parity status, menopausal status, presenting stage, or rate of cancer recurrence. Figure 4 presents the probability of PFS among the 57 women with type II tumors. The median PFS of the women with high expression of PD-L1 was 21 months which did not differ from that of the women with low expression (median PFS: 24 months).

After excluding 40 women who received NACT, the median PFS of women with high PD-L1 expression was 28 months compared to 62 months in the women with low expression (Figure 5). In the 62 women with type I tumors who did not receive neoadjuvant chemotherapy, the median PFS of those with high PD-L1 expression was 24 months, whereas it was not reached in the women with low expression (Figure 6). Figure 7 presents the probability of PFS among 30 women with type II tumors who did not receive NACT. There was no significant difference in PFS among the two comparison groups.

## Discussion

Overall, the surgical specimens from approximately 63% of the cases in this study exhibited high PD-L1 expression. There was no difference in the rate of high expression of PD-L1 between the two types of EOC. In type I tumors, high expression of PD-L1 was associated with later stages, disease recurrence, and shorter median PFS. In type II tumors, there was no an apparent difference between high and low expression of PD-L1 in terms of presenting stage, tumor recurrence, or survival. To our knowledge, this is the first report investigating the prognostic significance of PD-L1 expression stratified by type of EOC in order to avoid the effects of heterogeneous disease characteristics. 

Published data regarding the prognostic value of PD-L1 expression in type I EOC specifically is extremely limited. A study by Zhu et al., (2017) conducted in 122 women with ovarian clear cell carcinoma was the first report to evaluate the prognostic significance of PD-L1 expression. The study noted that high expression of PD-L1 was associated with advanced stages, recurrence, and poorer survival. An important finding of our study is the prognostic significance of PD-L1 status on the treatment outcomes in type I EOC. Women with high PD-L1 expression in our study were more likely to present at advanced stages and experience disease recurrence than those with low expression (51.0% versus 34.6% and 46.9% versus 26.9%, respectively). The median PFS of women with high PD-L1 expression was also shorter than that of those with low expression (27 months versus 62 months; Figure 3). Future studies are warranted to confirm these interesting results and to assess the potential role of PD-L1 checkpoint blockade therapy in type I EOC, particularly in ovarian clear cell carcinoma and mucinous carcinoma, which are relatively resistant to conventional chemotherapy regimens (Del Carmen et al., 2012; Winter et al., 2007).

Previous studies are contradictory as to whether the PD-L1 status of cancer cells is a helpful predictor for treatment outcomes in cases of type II EOC. Wang et al., (2017) noted that survival rate of patients diagnosed with high-grade serous ovarian carcinoma who had positive PD-L1 expression was significantly lower than that of those with negative expression. By contrast, another study by Darb-Esfahani et al., (2016) reported a favorable prognoses in cases of high-grade serous ovarian cancer that had high PD-L1 expression. However, our study found no prognostic impact of PD-L1 expression for type II EOC, which is in line with a report by Mesnage et al., (2017), in which more than 80% of their samples were type II EOC. There were no differences between women with high PD-L1 expression and those with low expression in terms of presenting stage and cancer recurrence. Moreover, the median PFS of women with high expression of PD-L1 did not differ from that of those with low expression (21 months versus 24 months). Further investigation is needed to clarify these conflicting results.

Previous studies have yielded inconclusive results regarding the impact of NACT on expression of PD-L1 (Lim et al., 2016; Pelekanou et al., 2017; Remark et al., 2016; Richter et al., 2017; Song et al., 2016). Some studies reported increases in PD-L1 expression following NACT compared to pretreatment specimens in patients with squamous cell carcinoma of the lung and esophagus (Lim et al., 2016; Song et al., 2016). By contrast, other studies did not find any changes in the PD-L1 expression after NACT in advanced-stage non-small cell lung cancer or advanced rectal adenocarcinoma (Remark et al., 2016; Richter et al., 2017). Interestingly, a previous study conducted among breast cancer patients noted that PD-L1 expression had decreased compared to pretreatment samples in cases of residual disease (Pelekanou et al., 2017). 

Few publications have described altered expression of PD-L1 as a result of NACT exposure in patients with EOC (Bohm et al., 2016; Mesnage et al., 2017). In a subset of 27 women with EOC who had paired pre- or post-NACT specimens, the rate of PD-L1-positive cases (using a cutoff of ≥5%) had increased from 30% to 63%. Furthermore, 63% of the cases that were PD-L1-negative had become to be positive following NACT (Mesnage et al., 2017). In a study conducted in 54 patients with metastatic high-grade serous carcinoma of the fallopian tube or ovary, PD-L1 levels were remarkably higher following NATC (Bohm et al., 2016). The increased expression of PD-L1 following NACT suggest that sequential chemoimmunotherapy may have a potential role in EOC treatment (Bohm et al., 2016). In this study, approximately 30% of women received NACT. However, we were unable to determine the impact of NACT on PD-L1 expression as all materials of tumors from the women who had received NACT were obtained after NACT. This may raise concern regarding whether NACT affected the findings in this study. However, the prognostic significances of PD-L1 expression on survival among the two types of epithelial ovarian cancer were unchanged after excluding the patients who had received NACT. Women with type I tumors and high PD-L1 expression had a significantly shorter PFS than those with low expression, but this was not true of women with type II tumors (Figures 6 and 7).

In the present study, type II tumors presented in advanced stages in approximately 80% of case compared to 45% of type I tumors. However, there was remarkably less tumor burden in the ovary in type II tumors, thus indicating early spreading. Unsurprisingly, women with type II tumors carried a higher rate of cancer recurrence than those with type I tumors (Table 1). This study also observed that women with type II tumors were significantly older, and that these tumors were more likely to occur in multiparous women than type I tumors (Table 1). These findings were in line with the model of the origin and pathogenesis of EOC that was revised by Kurman and Shih, (2016). 

Some limitations of this study are worthy of note. Given the relatively short follow-up time, we only used PFS as a meaningful endpoint for survival. Another limitation is the small sample size, which precluded the use of multivariate analysis for determining the independent effect of PD-L1 expression. The relatively small study size also hampered subgroup analysis in assessing the impact of PD-L1 status on each histologic subtype of type I EOC. In spite of these limitations, our study provides valuable insights into the prognostic value of PD-L1 expression in type I EOC. Further studies should be conducted to confirm this promising result and to determine the therapeutic potential of therapies targeted towards the PD-L1 signaling pathway in cases of type I EOC.

In conclusion, approximately 63% of EOC cases in this study were observed to have high PD-L1 expression. There was no significant difference in terms of expression of PD-L1 between the two types of EOC. Women with type I tumors who had high-intensity PD-L1 expression had poorer prognoses than those with low expression, but this was not true in women with type II tumors. 

## Statement conflict of Interest

None known.
